# Dopamine D2 Receptor Is Involved in Alleviation of Type II Collagen-Induced Arthritis in Mice

**DOI:** 10.1155/2015/496759

**Published:** 2015-11-29

**Authors:** Jian-Hua Lu, Yi-Qian Liu, Qiao-Wen Deng, Yu-Ping Peng, Yi-Hua Qiu

**Affiliations:** Department of Physiology, School of Medicine and Co-Innovation Center of Neuroregeneration, Nantong University, 19 Qixiu Road, Nantong, Jiangsu 226001, China

## Abstract

Human and murine lymphocytes express dopamine (DA) D2-like receptors including DRD2, DRD3, and DRD4. However, their roles in rheumatoid arthritis (RA) are less clear. Here we showed that lymphocyte DRD2 activation alleviates both imbalance of T-helper (Th)17/T-regulatory (Treg) cells and inflamed symptoms in a mouse arthritis model of RA. Collagen-induced arthritis (CIA) was prepared by intradermal injection of chicken collagen type II (CII) in tail base of DBA/1 mice or* Drd2* ^−/−^ C57BL/6 mice. D2-like receptor agonist quinpirole downregulated expression of proinflammatory Th17-related cytokines interleukin- (IL-) 17 and IL-22 but further upregulated expression of anti-inflammatory Treg-related cytokines transforming growth factor- (TGF-) *β* and IL-10 in lymphocytes* in vitro* and in ankle joints* in vivo* in CIA mice. Quinpirole intraperitoneal administration reduced both clinical arthritis score and serum anti-CII IgG level in CIA mice. However,* Drd2* ^−/−^ CIA mice manifested more severe limb inflammation and higher serum anti-CII IgG level and further upregulated IL-17 and IL-22 expression and downregulated TGF-*β* and IL-10 expression than wild-type CIA mice. In contrast,* Drd1* ^−/−^ CIA mice did not alter limb inflammation or anti-CII IgG level compared with wild-type CIA mice. These results suggest that DRD2 activation is involved in alleviation of CIA symptoms by amelioration of Th17/Treg imbalance.

## 1. Introduction

Dopamine (DA), a neurotransmitter in the central nervous system (CNS), has been found in the immune system [1, 2]. Both thymus and spleen express a dopaminergic system characterized by the presence of DA, vesicular monoamine transporters, and five subtypes of DA receptors [3]. The presence of these dopaminergic markers suggests that DA likely originating from immune cells and/or from sympathetic neuroeffector plexus is released in the lymphoid microenvironment [3]. Our previous work has shown that lymphocytes synthesize and secrete DA, and in turn it modulates lymphocyte function via autoendocrine and/or paraendocrine pathways [4–8]. Receptors of DA are essential for its regulation of cell function. The five subtypes of DA receptors, dopamine D1 receptor (DRD1) to DRD5, have been identified. On the basis of biochemical and pharmacological properties, DRD1 and DRD5 are attributed to D1-like receptors, and DRD2, DRD3, and DRD4 belong to D2-like receptors [9–11]. Human and murine lymphocytes express all the five subtypes of DA receptors [2, 6, 12–17]. Stimulation of different subtypes of DA receptors activates distinct intracellular signaling pathways and hence DA exerts different regulatory effects on lymphocytes. Studies carried out on human and murine T cells have shown that stimulation of D1-like receptors impairs T cell function by causing the rise of intracellular cAMP levels [1]. For example, stimulation of D1-like receptors impairs function and differentiation of T-regulatory (Treg) cells [16, 18] and promotes polarization of naive CD4^+^ T cells toward T-helper (Th)17 cells [17]. However, activation of DRD2 increases production of interleukin- (IL-) 10, a cytokine that negatively regulates function of effector T cells, which is involved in the polarization toward Treg cells [19]. Because Th17 and Treg cells are involved in autoimmunity as autoaggressive and beneficial cells, respectively, it is likely that D1-like and D2-like receptors expressed on T cells are involved in the interface between autoimmunity and health [1].

Rheumatoid arthritis (RA) is a representative human autoimmune disease characterized by systemic disorder as well as joint inflammation, destruction, and deformity [20, 21]. Among various immune cells, CD4^+^ T cells are critically implicated in pathogenesis of RA [22]. CD4^+^ T cells can differentiate into the several subsets, Th1, Th2, Th17, and Treg. Previously, RA was considered to be a purely Th1-mediated disease [22]. This classic paradigm was maintained until 2005, when a distinct lineage of proinflammatory Th17 cells was identified [23, 24]. Th17 cells are characterized by production of proinflammatory cytokines, such as IL-17 and IL-22. Currently, Th17 cells and their proinflammatory cytokines have been shown to significantly contribute to RA development [25, 26]. In addition to Th17 cells, Treg cells have been highlighted in both pathogenesis of RA and therapeutic strategies for RA [27, 28]. Treg cells suppress autoimmune processes and maintain peripheral tolerance by producing anti-inflammatory cytokines such as transforming growth factor- (TGF-) *β* and IL-10 [28–30]. Therefore, a major role in the pathogenesis of RA is attributed to immune dysregulation depending on the imbalance between anti-inflammatory Treg cells and proinflammatory Th17 cells [31, 32]. Thus, inhibiting Th17/Treg imbalance is potently promising to alleviate RA development [33]. However, it is unclear whether DRD2 activation alleviates Th17/Treg imbalance and thereby ameliorates RA development.

Accordingly, here we employed type II collagen-induced arthritis (CIA), a most common and reliable mouse model of RA, to elucidate DRD2 role in this autoimmune disease. CIA has the clinical symptoms and pathological changes similar to RA and therefore is widely used to investigate human RA pathogenesis and to explore new therapeutic strategies [34]. In the present study, we firstly determined that D2-like receptor agonist quinpirole downregulated Th17 proinflammatory cytokine expression but upregulated Treg anti-inflammatory cytokine expression in CIA mice* in vitro*; secondly we established that this agonist quinpirole ameliorated clinical symptoms and Th17/Treg imbalance* in vivo* in CIA mice; and lastly we demonstrated that* Drd2*-knockout (*Drd2* ^−/−^) mice had more severe arthritic symptoms and Th17/Treg imbalance in CIA process. These findings provide a new clue with DRD2 as a therapeutic target for RA.

## 2. Materials and Methods

### 2.1. Mice

Male DBA/1 mice (8–10 weeks, weighing 20–25 g) were obtained from Center of Experimental Animals, Nantong University, China.* Drd2* (B6.129S2-*Drd*2^tm1Low^/J) and* Drd1* (B6.129S4-*Drd*1*a* ^tm1Jcd^/J) null mice were kindly provided by Drs. Y. Q. Ding and J. W. Zhou, Shanghai Institutes for Biological Sciences, Chinese Academy of Sciences.* Drd2* ^−/−^ mice in a C57BL/6 (inbred) genetic background generated by 10 backcrosses were used. Animals were housed in a temperature- and light-controlled room (22°C, 12/12 h light/dark cycle) with food and water provided ad libitum. Animal experiments done in this study followed the policy guidelines of the National Institute of Health Guide for the Care and Use of Laboratory Animals (NIH publications no. 80-23) revised 1996.

### 2.2. Induction of CIA

CIA was induced for DBA/1 mice as described previously [35, 36] with small modifications. Briefly, chicken collagen type II (CII, Sigma-Aldrich Co., USA) was dissolved at a concentration of 2 mg/mL in 0.1 M acetic acid and emulsified with an equal volume of complete Freund's adjuvant (Chondrex, Inc., USA). As a primary immunization, 0.1 mL of the emulsion containing 100 *μ*g CII was intradermally injected into the tail base of mice on day 0. On day 21, the mice were intraperitoneally boosted with 100 *μ*g CII in incomplete Freund's adjuvant (Sigma-Aldrich Co., USA). Eventually, an intraperitoneal injection of 20 *μ*g LPS dissolved in 20 *μ*L PBS was executed to the mice on day 28 [37].

CIA induction for* Drd2* ^−/−^ mice was a little different from that for DBA/1 mice, as* Drd2* ^−/−^ mice were C57BL6 background. On day 21 after primary immunization that was the same as DBA/1 mice, C57BL6 mice received both intraperitoneal injection with 60 *μ*g CII in incomplete Freund's adjuvant and two hind-footpad injections with 40 *μ*g CII, based on [35, 38].

### 2.3. Evaluation of CIA

Clinical symptoms and arthritis scores of mouse limbs were observed by two independently blinded examiners every other day from day 21 after primary immunization. The clinical arthritis scores were evaluated using a scale of 0–3 for each limb: grade 0, no swelling; grade 1, slight swelling and erythema; grade 2, pronounced swelling; and grade 3, severe swelling and/or joint rigidity [39–41]. The average score was expressed as a cumulative value for all paws, with a maximal possible score of 12 for each animal. Before mice were killed on day 41 after immunization, microcaliper was used to measure the thickness of hind paws and the width of ankle joints.

### 2.4. Treatment of CIA

DBA/1 mice were divided into 3 groups, intact, CIA + vehicle, and CIA + quinpirole. The selective D2-like receptor agonist quinpirole (Santa Cruz Biotechnology, Inc., USA), which was dissolved in normal sodium, was administered (0.3 mg/kg) intraperitoneally twice a week for three weeks beginning from day 18 after primary immunization. Mice in the vehicle group received equal volume of normal sodium.

### 2.5. Enzyme-Linked Immunosorbent Assay (ELISA) for Measurement of Anti-CII IgG Level in the Serum

Serum from mice was collected and stored at −80°C until assayed. Anti-CII IgG antibody level was measured by ELISA. In brief, 96-well flat-bottomed microtiter plates were incubated with 100 *μ*L/well of CII (100 *μ*g/mL) at 4°C overnight and washed three times with PBS containing 0.05% Tween 20. The wells were then blocked by incubation with 100 *μ*L of PBS containing 1% ovalbumin (Sigma, USA) at 37°C for 1 h. After washing, the plates were incubated with 100 *μ*L of 1 : 1,000 dilution of each serum sample at 37°C for 30 min. The plates were washed, and 100 *μ*L/well of 1 : 5,000 dilution of goat anti-mouse IgG labeled with horseradish peroxidase (Invitrogen, USA) was added and incubated at 37°C for 1 h. After washing, 100 *μ*L/well 3,3′5,5′-tetramethylbenzidine was added to each well, which was incubated for 10 min. The reaction was stopped with 100 *μ*L H_2_SO_4_. Absorbance was determined with a multimode microplate reader (Bio Tek, USA) at 450 nm.

### 2.6. Culture and Treatment of Splenic Lymphocytes

On the 41st day after primary immunization, the spleens were harvested from the anaesthetized DBA/1 mice by celiotomy. Lymphocyte cultures were prepared as described previously [42, 43] with small modifications. Briefly, single cell suspensions were obtained by gently squeezing the spleens in RPMI 1640 culture medium (Gibco, USA) and passing the tissue through a 200 *μ*m nylon mesh screen. Splenic mononuclear cells were isolated from single cells by Ficoll-Hypaque density gradient centrifugation (specific gravity: 1.080, 2000 rpm, 30 min). Mononuclear cells were taken from the intermediate layer and washed three times with RPMI 1640 culture medium. And then the splenic mononuclear cells were resuspended in complete culture medium at a concentration of 1.8 × 10^6^ cells/mL. Quinpirole was added to the culture at a final concentration of 10^−6^ M. After 30 minutes, concanavalin A (Con A, Sigma) was also added at a final concentration of 5 *μ*g/mL. Ultimately the cultures were incubated in the incubator (ESPEC BNA-311, Japan) with 5% CO_2_ at 37°C for 48 h.

### 2.7. Western Blot Analysis

Proteins were extracted from splenic lymphocytes or from pulverized ankle joints with ultrasound in lysis buffer and the supernatants were collected by centrifuging at 4°C at 12,000 ×g for 15 min. Equal amount of proteins was loaded onto a 12% sodium dodecyl sulfate-polyacrylamide gel and subjected to electrophoresis. And then the proteins were transferred onto a polyvinylidene difluoride membrane (Pall, USA). After blocking nonspecific binding with 5% skim milk, the membranes were probed with mouse antibodies specific for DRD2 (1 : 200, Invitrogen, USA), IL-10 (1 : 200, Santa Cruz Biotechnology, Inc., USA), or *β*-actin antibody (1 : 1,000, Sigma-Aldrich Co., USA) or with rabbit antibodies specific for DRD1 (1 : 200, Invitrogen, USA), IL-17 (1 : 200, Santa Cruz Biotechnology, Inc., USA), TGF-*β* (1 : 300, Abcam, UK), or IL-22 (1 : 200, Santa Cruz Biotechnology, Inc., USA) at 4°C overnight. Then, the membranes were incubated with the IRDye 700-conjugated affinity-purified goat anti-mouse IgG (1 : 5,000, Rockland Immunochemicals, USA) or with IRDye 800-conjugated affinity-purified goat anti-rabbit IgG (1 : 5,000, Rockland Immunochemicals, USA) for 1 h at room temperature, followed by visualization using Odyssey laser scanning system (LI-COR Inc., USA). The relative quantity of the protein bands was determined by an image analysis system (Odyssey 3.0 software).

### 2.8. Statistical Analysis

Data were expressed as mean ± standard deviation (M ± SD). Statistical analyses were performed with the Statistics Package for Social Science (SPSS, 19.0). The data were subjected to one-way analysis of variance (ANOVA), followed by Student-Newman-Keuls test, to compare the data of all groups relative to each other. Statistical significance was set at *p* < 0.05.

## 3. Results

### 3.1. DRD2 Expression Increases and D2-Like Receptor Agonist Quinpirole Downregulates Th17-Related Cytokine Expression but Upregulates Treg-Related Cytokine Expression in Lymphocytes in CIA Mice

**Figure 1:**
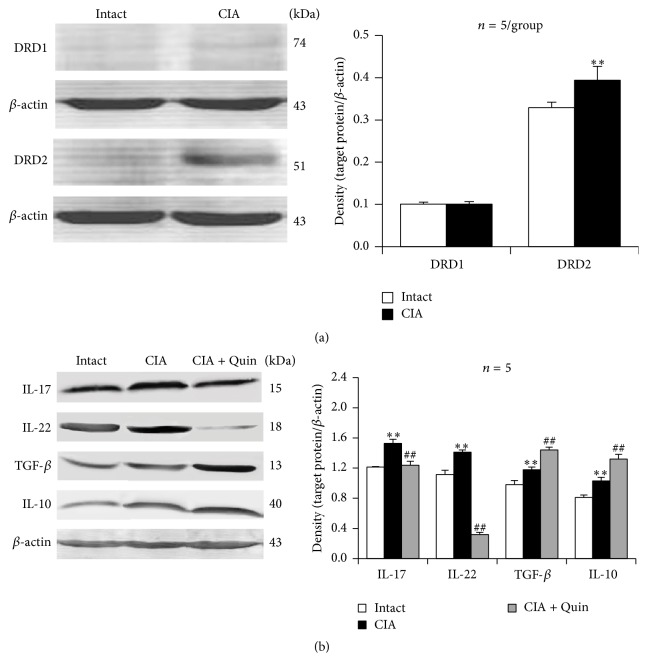
DRD2 expression increases and D2-like receptor agonist quinpirole downregulates Th17-related cytokine expression but upregulates Treg-related cytokine expression in lymphocytes in CIA mice. Lymphocytes from spleens of CIA mice (at day 41 after primary immunization) were exposed to quinpirole in the presence of Con A for 48 h. Proteins were extracted and Western blot analysis was performed. (a) DRD1 and DRD2 expression in Con A-activated lymphocytes. (b) Left panel shows representative bands and right panel is a statistical graph. Quin: quinpirole; ^*∗∗*^ *p* < 0.01 versus intact; ^##^ *p* < 0.01 versus CIA.

To show an involvement of DRD2 in CIA pathogenesis, we firstly examined DRD2 expression in lymphocytes from spleens of CIA mice. The splenic lymphocytes that were stimulated by Con A expressed more DRD2 in CIA mice than in intact mice (Figure 1(a)). As a control, DRD1 expression was not altered in the lymphocytes of CIA mice compared with intact mice (Figure 1(a)). Moreover, the expression of both the proinflammatory Th17-related cytokines IL-17 and IL-22 and the anti-inflammatory Treg-related cytokines TGF-*β* and IL-10 was upregulated in CIA lymphocytes relative to that in intact lymphocytes in the presence of Con A (Figure 1(b)). Importantly, the D2-like receptor agonist quinpirole remarkably downregulated IL-17 and IL-22 expression but further upregulated TGF-*β* and IL-10 expression in CIA lymphocytes with respect to quinpirole-untreated CIA lymphocytes (Figure 1(b)).

### 3.2. D2-Like Receptor Agonist Quinpirole Alleviates Arthritic Symptoms and Downregulates Th17-Related Cytokines and Upregulates Treg-Related Cytokines* In Vivo* in CIA Mice

**Figure 2:**
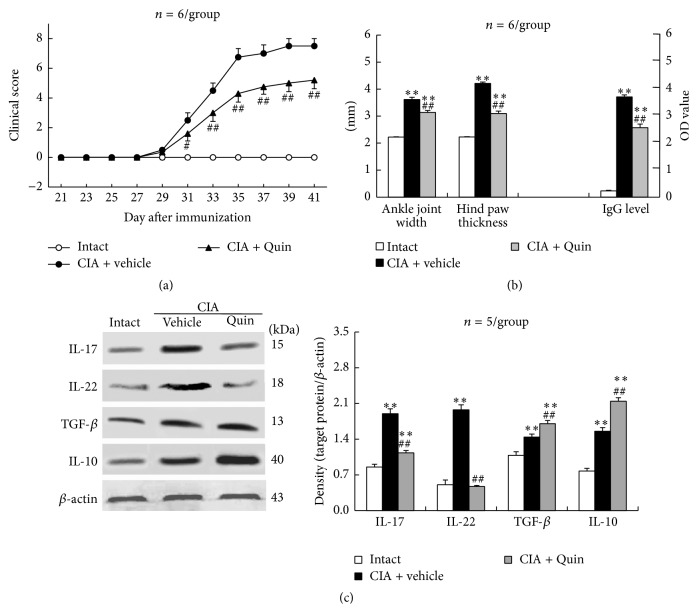
D2-like receptor agonist quinpirole alleviates clinical symptoms and downregulates Th17-related cytokines and upregulates Treg-related cytokines in ankle joints in CIA mice. Mice were intraperitoneally administered with the D2-like receptor agonist quinpirole twice a week for three weeks dating from day 18 after primary immunization. The clinical arthritis score of four limbs was observed every other day beginning from day 21 until day 41 after immunization (a). The ankle joint width, hind paw thickness, and serum anti-CII IgG titer were measured on day 41 after immunization (b). Protein expression of the cytokines in ankle joints was examined on day 41 after immunization (c). Quin: quinpirole; ^*∗∗*^ *p* < 0.01 versus intact; ^#^ *p* < 0.05, ^##^ *p* < 0.01 versus CIA + vehicle.

Mice were intraperitoneally administered with the D2-like receptor agonist quinpirole twice a week for three weeks dating from day 18 after primary immunization. Arthritic symptoms were assessed by clinical arthritis score of four limbs, width of two ankle joints, and thickness of two hind paws. The clinical arthritis score was recorded every other day beginning from day 21 until day 41 after immunization. A notable rise of clinical arthritis score started on day 31 after immunization, reached almost the peak on day 35, and kept at the highest value (7.5) until day 41 (Figure 2(a)). The quinpirole treatment of CIA mice significantly reduced clinical arthritis score at observed time points during days 31 through 41 after immunization with respect to vehicle-treated CIA mice (Figure 2(a)). In addition, compared with intact animals, CIA mice manifested a remarkable increase in ankle joint width and hind paw thickness, which were measured on day 41 after immunization (Figure 2(b)). The quinpirole injection significantly decreased the ankle joint width and hind paw thickness of CIA mice, although the inflammatory parameters did not drop down to intact levels (Figure 2(b)). In addition to the inflammatory manifestations of arthritis, serum anti-CII IgG level was notably elevated on day 41 after immunization (Figure 2(b)). The quinpirole administration significantly reduced serum anti-CII IgG level in CIA mice relative to vehicle treatment (Figure 2(b)).

Notably, the upregulated expression of IL-17 and IL-22 in ankle joints of CIA mice was dramatically reduced by quinpirole treatment, whereas the upregulated expression of TGF-*β* and IL-10 was significantly enhanced by quinpirole treatment (Figure 2(c)).

### 3.3. *Drd2* Deficiency Aggravates CIA Clinical Symptoms and Proinflammatory Cytokine Upregulation

**Figure 3:**
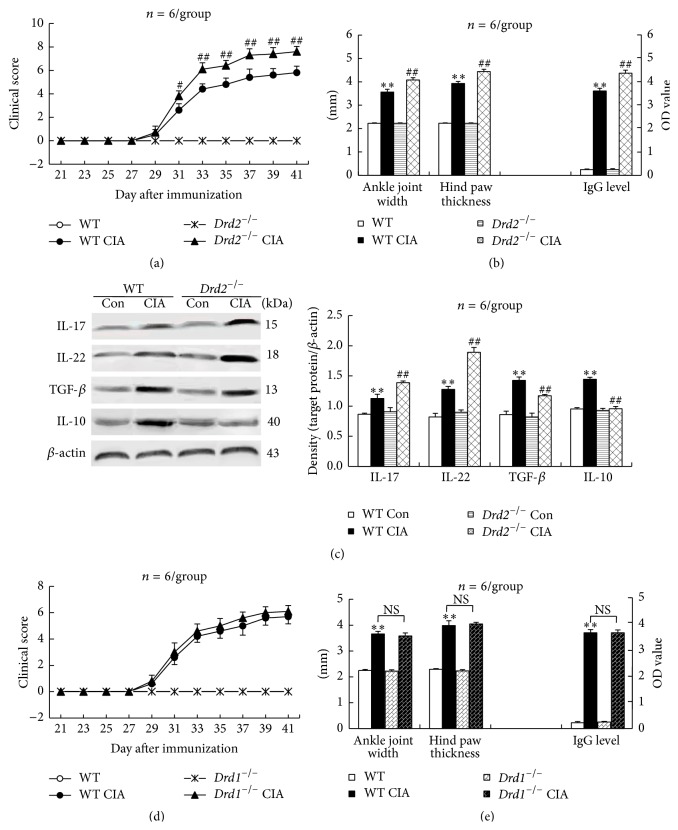
*Drd2* deficiency aggravates CIA clinical symptoms and proinflammatory cytokine upregulation. This experimental design was similar to that of Figure 2, except that* Drd2*-deficient mice (no quinpirole treatment) and* Drd1*-deficient mice were used instead. Con: control; WT: wild-type mice; NS: no significance; ^*∗∗*^ *p* < 0.01 versus WT or WT Con; ^#^ *p* < 0.05, ^##^ *p* < 0.01 versus WT CIA.

Wild-type mice manifested a continuously increased clinical arthritis score beginning at day 31 until day 41, the last day observed, after primary immunization (Figure 3(a)). Likewise, CII induced a significant increase in ankle joint width, hind paw thickness, and serum anti-CII IgG antibody level in wild-type mice on day 41 after immunization (Figure 3(b)). Importantly,* Drd2*-knockout mice manifested more severe inflammation of limbs as determined by the increased clinical arthritis score, ankle joint width, and hind paw thickness, as well as higher serum anti-CII IgG level than wild-type mice (Figures 3(a) and 3(b)).

In addition,* Drd2*-null mice showed further upregulated IL-17 and IL-22 expression in ankle joints compared with wild-type mice on day 41 after primary immunization with CII antigen (Figure 3(c)). However, the upregulated TGF-*β* and IL-10 expression induced by CII antigen in wild-type mice was reduced (TGF-*β*) or disappeared (IL-10) in* Drd2* ^−/−^ mice (Figure 3(c)). Moreover,* Drd2*-deficient mice had neither arthritic manifestations nor proinflammatory/anti-inflammatory cytokine disorders in the absence of CII antigen induction, as wild-type mice did (Figures 3(a), 3(b), and 3(c)).

Furthermore,* Drd1*-knockout mice were induced for CIA and measured for CIA symptoms. Unlike* Drd2* ^−/−^ CIA mice,* Drd1*-null CIA mice did not manifest either more severe inflammation of limbs as determined by clinical arthritis score, ankle joint width, and hind paw thickness or higher serum anti-CII IgG level, in comparison with wild-type CIA mice (Figures 3(d) and 3(e)).

## 4. Discussion

In the current study, lymphocytes from the spleens of CIA mice had an upregulated expression of IL-17 and IL-22 in response to Con A. In addition, in the inflamed ankle joints in CIA mice, IL-17 and IL-22 expression was upregulated. These data support an enhanced Th17-cell proinflammatory activity in CIA process. Interestingly, the Treg-related anti-inflammatory cytokines, TGF-*β* and IL-10, were also upregulated both in splenic lymphocytes* in vitro* and in inflamed ankle joints* in vivo* in CIA mice. These results imply an increased Treg cell anti-inflammatory activity in CIA process. The enhanced Th17 cell activity, including increased Th17 frequency and increased IL-17 and IL-22 release in peripheral blood, has been associated with RA pathogenesis [32, 44–46]. However, the changes in Treg cell activity in RA patients are not so consistent. For example, Treg frequency is decreased in peripheral blood of RA patients [32, 44–46], but Treg immunosuppressive function [45] and IL-10 release from peripheral blood mononuclear cells (PBMCs) in response to LPS [44] are not significantly altered in RA patients. Interestingly, a higher level of serum IL-4, IL-5, and IL-10 is found in both inactive and active RA patients than in healthy individuals [32], suggesting an enhanced anti-inflammatory activity in RA. Collectively, these findings suggest that Th17 proinflammatory activity is definitely enhanced in RA as a cause or/and a result of RA, but Treg anti-inflammatory activity in RA is indefinite, depending on disease severity, inflammatory strength, and individual response difference in RA occurrence and progression. Our present results support an enhanced Treg function in CIA. The increased anti-inflammatory activity may represent a compensative protection against further joint inflammation and injury. Nevertheless, the upregulated Treg-related cytokine expression was not so prominent as the upregulated Th17-related cytokine expression. Thus, an imbalance of Th17/Treg cell function at a higher level is suggested in CIA inflammatory circumstance.

Notably, DA D2-like receptor agonist quinpirole dramatically downregulated IL-17 and IL-22 expression and further upregulated TGF-*β* and IL-10 expression in lymphocytes* in vitro* in CIA mice. These data strongly show that quinpirole suppresses Th17 cell proinflammatory response and enhances Treg-cell anti-inflammatory response. As a result, a new balance of Th17/Treg cell function or even a functional bias toward Treg cells appears in CIA by the quinpirole treatment. These changes help to promote CIA recovery. As expected, quinpirole administration* in vivo* alleviated inflammatory manifestations, inhibited Th17 proinflammatory response, and enhanced Treg anti-inflammatory activity in CIA. These consistent results* in vitro* and* in vivo* demonstrate that D2-like receptor activation is beneficial to amelioration of CIA. Nakano et al. [47] have shown that, in RA synovial/SCID mouse chimera model, a selective D2-like receptor antagonist haloperidol significantly induces accumulation of IL-6^+^ and IL-17^+^ T cells with exacerbated cartilage destruction. The opposite effects of D2-like receptor agonist in this study to those of D2-like receptor antagonist in Nakano et al. study provide substantial evidence for the beneficial role of D2-like receptors in CIA disease.

DA D2-like receptors include DRD2, DRD3, and DRD4 that are expressed on human and murine T lymphocytes [2, 6, 48]. Although the D2-like receptor agonist quinpirole is of higher affinity to DRD2, it can also activate DRD3 [49]. To further identify the role of DRD2 in CIA, we employed* Drd2*-knockout mice.* Drd2*-deficient CIA mice manifested more severe inflamed symptoms of limbs and further elevated serum anti-CII IgG level as well as joint Th17-proinflammatory cytokine expression than wild-type CIA mice. However, expression of the anti-inflammatory cytokines TGF-*β* and IL-10 in ankle joints of* Drd2*-deficient CIA mice was remarkably downregulated compared with that of wild-type CIA mice. These findings demonstrate that* Drd2* deficiency exacerbates CIA inflamed manifestations and imbalance of Th17/Treg cell function. In contrast,* Drd1*-deficient CIA mice did not alter either the inflamed symptoms of limbs or the increased serum anti-CII IgG level in comparison with wild-type CIA mice. In support of these results, we found that DRD1 expression in lymphocytes was not altered in CIA mice, whereas DRD2 expression was upregulated in CIA mice. The findings identify an involvement of DRD2 but not DRD1 in CIA disease. Dissimilarly, in RA synovial/SCID mouse chimera model, a selective D1-like receptor antagonist SCH-23390 alleviates both accumulation of IL-6^+^ and IL-17^+^ T cells and cartilage destruction [47], suggesting an involvement of D1-like receptors in RA disease. Since the antagonist SCH-23390 can also block DRD5 action [50], the effects caused by SCH-23390 may include the consequence of DRD5 blockage. On the other hand, the RA synovial/SCID mouse chimera model is not quite the same with the CIA model in pathogenesis or/and manifestations. The two aspects of reasons may contribute to the different effects between* Drd1*-knockout and D1-like receptor antagonist on CIA or RA diseases.

Interestingly, DRD2 expression on lymphocytes in CIA mice was upregulated in the present study. As a support, Nakano et al. [47] have reported that DA is significantly increased in RA synovial fluid and localized in dendritic cells (DCs) in the synovial tissue of RA patients. These changes in CIA or RA diseases may represent a compensatory protection mechanism against further deterioration of the diseases. This explanation is supported by the finding that the level of DRD2 expression on lymphocytes in RA patients negatively correlates with the disease activity [51]. In addition, the same authors [51] found that DRD2 expression level is lower on lymphocytes in RA patients than in healthy individuals. This seems to be inconsistent with our present result showing that DRD2 expression on lymphocytes in CIA mice was increased. The inconsistence is probably attributed to different disease stages and disease severity. Further investigation is needed to elucidate this problem. Collectively, a novel therapeutic strategy aimed at stimulating DRD2 system in lymphocytes is suggested to be potentially promising for inhibition of RA development.
